# A Dual-Antigen SARS-CoV-2 Serological Assay Reflects Antibody Avidity

**DOI:** 10.1128/jcm.02262-21

**Published:** 2022-02-16

**Authors:** Yu Nakagama, Yuko Nitahara, Natsuko Kaku, Evariste Tshibangu-Kabamba, Yasutoshi Kido

**Affiliations:** a Department of Parasitology, Osaka City University, Osaka, Japan; b Research Center for Infectious Disease Sciences, Graduate School of Medicine, Osaka City University, Osaka, Japan; Boston Children's Hospital

**Keywords:** avidity maturation, COVID-19, SARS-CoV-2, serological assay

## LETTER

Serial antibody measurements using an array of SARS-CoV-2 immunoassays have demonstrated differing kinetics among assay platforms (1). Immunoassays utilizing a double-antigen sandwich method (e.g., Roche Elecsys immunoassays) responded uniquely by reporting rising titer values along the longitudinal monitoring period. We wish to address the interpretation of these results with specific consideration of the avidity characteristics of the analyte.

**FIG 1:**
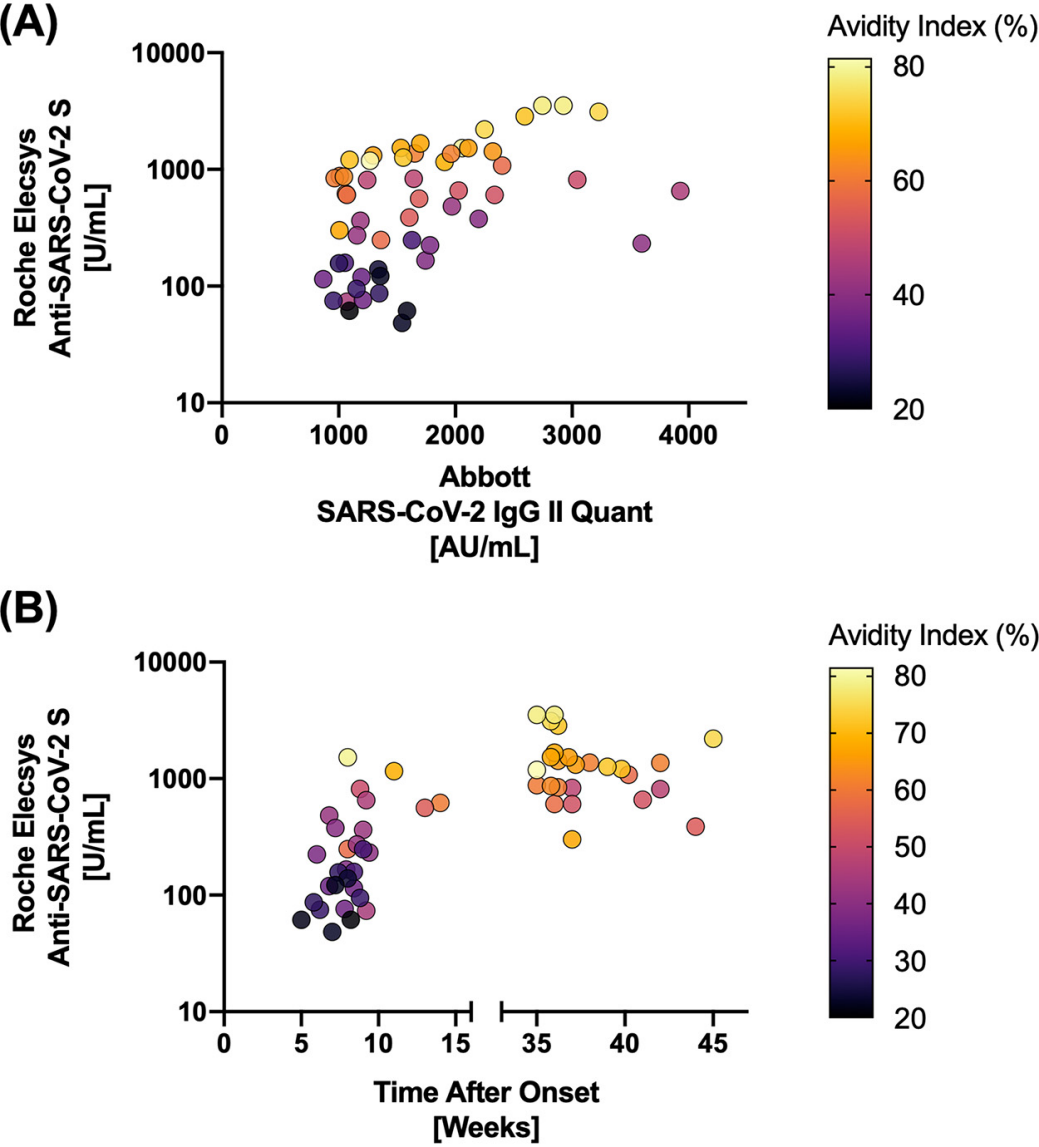
Roche Elecsys assay requires bivalent antigen binding and is tuned for high-avidity antibodies. (A) Antibodies of enhanced avidity return higher Roche Elecsys titer relative to the corresponding Abbott titer. (B) Sera from late convalescence demonstrate higher avidity and, hence, relatively higher Roche Elecsys titers.

In our present study, we evaluated a well-characterized subset of COVID-19 convalescent-phase sera (*n* = 55) for antibody titers targeting SARS-CoV-2 spike using Roche Elecsys anti-SARS-CoV-2 S (Roche, Basel, Switzerland) and Abbott SARS-CoV-2 IgG II Quant (Abbott, Chicago, IL, USA) immunoassays. Having been sampled across a wide range of times (2 to 10 months) postinfection, analyte sera harbored divergent maturity characteristics, as indicated in their avidity indices (median, 52.2%; interquartile range, 35.8 to 65.5%). Here, the avidity index was derived, per serum, by ratiometrically comparing optical density values obtained from the enzyme-linked immunosorbent assay (Euroimmun, Lübeck, Germany), performed with or without an extra 5.5 M urea treatment step (10 min at 37°C following incubation with spike antigen) (2). Sera of enhanced avidity returned consistently higher values for Roche Elecsys titer than Abbott titer (Fig. 1A). The degree of avidity maturation was associated with time after onset (Fig. 1B). Sera from late convalescence (range, 35 to 45 weeks from onset) demonstrated significantly higher avidity indices and, hence, relatively higher Roche Elecsys titers than those from early convalescence (range, 5 to 14 weeks from onset) (*P* < 0.0001, Mann-Whitney test). In contrast to two-step immunoassays using anti-human immunoglobulin for detection, the Roche Elecsys assay is based on the double-antigen sandwich method that, in principle, selectively targets bivalently bound antibodies. Roche Elecsys assay, by design, requires double-antigen binding for signal emission and carries an expected tendency toward detection of high-avidity antibodies with efficient binding capacity (3).

With the aim of harmonizing anti-SARS-CoV-2 immunity assessment, the World Health Organization (WHO) has released the international standard for anti-SARS-CoV-2 immunoglobulin (4). The universal binding antibody unit (BAU) enables cross-comparison among different assays by applying the assay-specific conversion factor suggested by each manufacturer, e.g., [BAU/ml] = [AU/ml] × (1/7) for Architect titer and [BAU/ml] = [U/ml] × 1 for Elecsys titer. The robustness of conversion factors under variable clinical scenarios, however, is questionable in light of these data.

In conclusion, our finding highlights the necessity of individualizing the interpretation of SARS-CoV-2 serological assay results depending on clinical context (i.e., the interval since recovery if convalescent, or, in the case of a vaccinee, the cumulative number of received doses), which critically affects the degree of avidity maturation of serum antibodies.

## References

[1] Theel ES, Johnson PW, Kunze KL, Wu L, Gorsh AP, Granger D, Roforth MM, Jerde CR, Lasho M, Andersen KJ, Murphy BM, Harring J, Lake DF, Svarovsky SA, Senefeld JW, Carter RE, Joyner MJ, Baumann NA, Mills JR. 2021. SARS-CoV-2 serologic assays dependent on dual-antigen binding demonstrate diverging kinetics relative to other antibody detection methods. J Clin Microbiol 59:e01231-21. 10.1128/JCM.01231-21. PMC8373029 34166066

[2] Pichler D, Baumgartner M, Kimpel J, Rössler A, Riepler L, Bates K, Fleischer V, von Laer D, Borena W, Würzner R. 2021. Marked increase in avidity of SARS-CoV-2 antibodies 7–8 months after infection is not diminished in old age. J Infect Dis 224:764–770. 10.1093/infdis/jiab300. 34086960 PMC8195195

[3] Muench P, Jochum S, Wenderoth V, Ofenloch-Haehnle B, Hombach M, Strobl M, Sadlowski H, Sachse C, Torriani G, Eckerle I, Riedel A. 2020. Development and validation of the Elecsys anti-SARS-CoV-2 immunoassay as a highly specific tool for determining past exposure to SARS-CoV-2. J Clin Microbiol 58:e01694-20. 10.1128/JCM.01694-20. 32747400 PMC7512151

[4] World Health Organization. 2021. First WHO international standard for anti-SARS-CoV-2 immunoglobulin and first 27 WHO International Reference Panel for anti-SARS-CoV-2 immunoglobulin, p 66–68. WHO technical 30 report series, no. 1030, section 9.1.2. World Health Organization, Geneva, Switzerland. https://www.who.int/publications/i/item/9789240024373.

